# Impaired APP activity and altered Tau splicing in embryonic stem cell-derived astrocytes obtained from an APPsw transgenic minipig

**DOI:** 10.1242/dmm.019489

**Published:** 2015-10-01

**Authors:** Vanessa J. Hall, Maiken M. Lindblad, Jannik E. Jakobsen, Anders Gunnarsson, Mette Schmidt, Mikkel A. Rasmussen, Daniela Volke, Thole Zuchner, Poul Hyttel

**Affiliations:** 1 Department of Veterinary Clinical and Animal Sciences, Faculty of Health and Medical Sciences, University of Copenhagen, DK-1870 Frederiksberg, Denmark; 2 Department of Veterinary Disease Biology, Faculty of Health and Medical Sciences, University of Copenhagen, DK-1870 Frederiksberg, Denmark; 3 Department of Biomedicine, Aarhus University, Faculty of Health, DK-8000 Aarhus, Denmark; 4 Department of Large Animal Sciences, Faculty of Life Sciences, University of Copenhagen, DK-1870 Frederiksberg, Denmark; 5 Bioneer Biomedical Technology, DK-2970 Hørsholm, Denmark; 6 Center for Biotechnology and Biomedicine, Institute of Bioanalytical Chemistry, University of Leipzig, 04103 Leipzig, Germany; 7 Octapharma Biopharmaceuticals GmbH, 69120 Heidelberg, Germany

**Keywords:** Alzheimer's disease, APP Swedish mutation, Pig, Radial glial cells, Astrocytes, Secretase expression, Tau phosphorylation, Cell cycle, Astrogenesis

## Abstract

Animal models of familial juvenile onset of Alzheimer's disease (AD) often fail to produce diverse pathological features of the disease by modification of single gene mutations that are responsible for the disease. They can hence be poor models for testing and development of novel drugs. Here, we analyze *in vitro*-produced stem cells and their derivatives from a large mammalian model of the disease created by overexpression of a single mutant human gene (*APPsw*). We produced hemizygous and homozygous radial glial-like cells following culture and differentiation of embryonic stem cells (ESCs) isolated from embryos obtained from mated hemizygous minipigs. These cells were confirmed to co-express varying neural markers, including NES, GFAP and BLBP, typical of type one radial glial cells (RGs) from the subgranular zone. These cells had altered expression of *CCND1* and *NOTCH1* and decreased expression of several ribosomal RNA genes. We found that these cells were able to differentiate into astrocytes upon directed differentiation. The astrocytes produced had decreased α- and β-secretase activity, increased γ-secretase activity and altered splicing of tau. This indicates novel aspects of early onset mechanisms related to cell renewal and function in familial AD astrocytes. These outcomes also highlight that radial glia could be a potentially useful population of cells for drug discovery, and that altered APP expression and altered tau phosphorylation can be detected in an *in vitro* model of the disease. Finally, it might be possible to use large mammal models to model familial AD by insertion of only a single mutation.

## Abstract (precis)

**Summary:** Insight into astrocyte and radial glia pathology in an *in vitro* culture system derived from the APPsw pig.

## INTRODUCTION

Alzheimer's disease (AD) is a progressive neurodegenerative disease that can occur sporadically or be inherited from familial mutations. The primary pathological lesions include deposition of extracellular amyloid plaques and neurofibrillary tangles composed of filamentous hyperphosphorylated tau protein. Neuronal loss, primarily in the cortex and CA1 portion of the hippocampus, is largely associated with degeneration of basal forebrain cholinergic neurons, with progressive cognitive and memory decline also occurring. In the case of the familial-inherited human amyloid precursor protein (APP) K670N-M671L Swedish mutation (APPsw), transgenic mice carrying this mutation develop amyloid β (Aβ) deposits by 11 months of age, gliosis and neuritic dystrophy, but not neuronal loss (Irizarry et al., 1997). Double PSAPP mutant mice expressing mutant presenilin 1 (PS1) and APPsw also show age-dependent Aβ deposition, but also no neuronal loss (Takeuchi et al., 2000). In addition, it has been shown that only when three transgenes, specifically, PS1, APPsw and tau P301L, are inserted into a mouse model, can Aβ, synaptic dysfunction and tangle pathology occur (Oddo et al., 2003). Despite the range of pathologies, hyperphosphorylation of tau was not observed in this model. Neuronal loss has, however, been reported in a rat model expressing both PS1 and APPsw (Cohen et al., 2013), indicating that some animal models might be more suitable at recapitulating the disease. Therefore, neurodegenerative diseases such as familial AD can be partially, but not fully, recapitulated in rodent models (Elder et al., 2010).

The development of new biomedical models of disease in large mammals indicates that they recapitulate the disease in many aspects more faithfully than rodents (Aigner et al., 2010; Luo et al., 2012). Transgenic pigs carrying human disease genes for familial inherited neurodegenerative disorders have also recently been produced, including for Huntington's disease (Yang et al., 2010) and AD (Kragh et al., 2009). In the latter study, the human APP containing the Swedish mutation in the 695 isoform under control of the PDGFβ promoter, was transfected into porcine Göttingen minipig fibroblasts which were used for somatic cell nuclear transfer and led to the generation of hemizygous animals. A follow-up study on one- to two-year-old hemizygous animals failed to show any cognitive decline by behavioral assessment (Søndergaard et al., 2012), and further comprehensive characterization of this model is required as these animals age. It is also unknown when a phenotype might be detectable, due to the relatively long lifespan of these animals (∼20 years). One alternative to waiting for the animals to age would be to study their cells *in vitro*. Deriving cell models of the disease is relatively easy in animal models and would be particularly useful for studying early basic cellular processes important in the development of the disease. Most importantly, it was of interest to us to determine whether this animal model would be better at recapitulating pathology related to AD compared with the current mouse APPsw models. In this study, we were able to produce radial glial-like cells (RGs) differentiated from embryonic stem cells (ESCs), which were isolated from D8 embryos generated from matings of hemizygous human APPsw (hAPPsw) minipigs. These cells were analyzed for potential defects in cell cycle and ribosome function. In addition, these cells could differentiate into astrocytes, which had altered secretase expression and impaired splicing of tau. Given the evidence that activated astrocytes cluster around plaques in the disease and that gliogenesis can be observed in some AD models (Bondolfi et al., 2002; Kamphuis et al., 2014), this model allows us to study an often neglected population studied in AD.

**TRANSLATIONAL IMPACT**

**Clinical issue**

Alzheimer's disease (AD) is a progressive neurodegenerative disease for which both sporadic and familial cases are known. One example of inherited mutation in humans is the amyloid precursor protein (APP) K670N-M671L Swedish mutation (APPsw). Transgenic mice carrying this mutation develop only a subset of the disease phenotypes that are associated with the human condition. The pig is a large mammal model that better recapitulates human neuropathology compared with rodent models, which has been demonstrated both anatomically and physiologically. Thus, this study aimed to determine whether transgenic Göttingen minipigs could be used as an animal model for recapitulating features of familial APPsw AD by overexpressing either a single copy or two copies of the human APPsw gene in these animals.

**Results**

An *in vitro* cell model of APPsw AD was established by culturing and differentiating embryonic stem cells isolated from the APPsw transgenic minipig. These cells expressed markers typical of Type 1 cells from the subgranular zone (SGZ) and were termed radial glia (RG)-like cells. These progenitors showed some features of early AD disease, including perturbations in some cell-cycle genes, such as *PTCH1* and *CCND1*, and decreased expression of ribosomal RNA genes. Unlike WT-cell-derived RGs (which can differentiate into neurons, oligodendrocytes or astrocytes), the RGs derived from APPsw transgenic minipig formed only astrocytes upon directed differentiation. The RG-derived astrocytes showed altered APP activity and altered splicing of tau (another protein involved in AD pathogenesis), providing new insights into the etiology of the disease in the white matter.

**Implications and future directions**

This study describes an *in vitro* cell culture system derived from a large mammal overexpressing the human APPsw mutation. This system revealed new insights into early defects of progenitor cells in familial APPsw AD and also helped to identify AD-related alterations in astrocytes derived from APPsw RGs. The RGs might be a useful source of cells for studying early AD mechanisms, which could also aid the identification of novel early targets of disease. In addition, the fact that astrocytes outnumber neurons fivefold in the brain underlines the importance of studying AD-related mechanisms in the white matter, an aspect that can be further investigated in this cell system. Finally, the scalability of *in vitro* cell-culture models of AD enables their use for screening large chemical compound libraries to identify new target compounds for drug development.

## RESULTS

### Production of RGs following differentiation of hAPPsw porcine ESCs

**Fig. 1.**
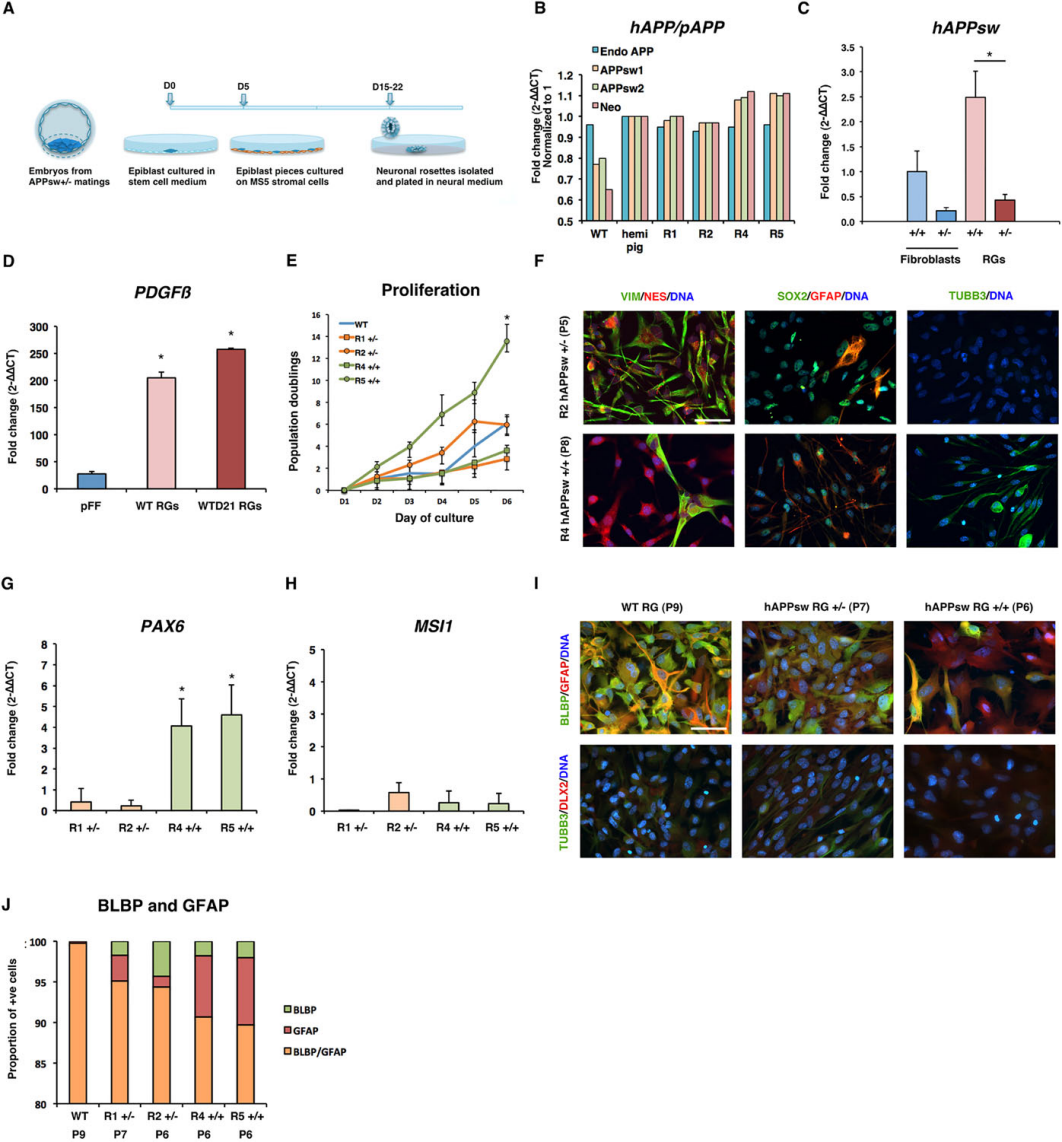
**Production and characterization of radial glial cells (RGs) from transgenic porcine embryonic stem cells, carrying the human APP Swedish mutation (hAPPsw).** (A) Overview of the procedure for the establishment of RGs. (B) Genotyping of lines using comparative qPCR revealed that two lines were hemizygous and two homozygous. (C) Expression of hAPPsw in hAPPsw^+/−^ and hAPPsw^+/+^ fibroblasts and RGs. Student's *t*-test was performed and significance was found when *P*≤0.05 (marked by *). (D) Expression of *PDGFβ* by comparative qPCR revealed increased expression in hAPPsw RGs and hAPPsw RG-differentiated astrocytes compared with non-transgenic porcine fetal fibroblasts (pFFs). Student's *t*-test was performed and significance was found when *P*≤0.05 (marked by *). (E) Population doublings of WT and hAPPsw RGs reveal that all lines proliferate at similar rates, except for R5 hAPPsw^+/+^ line, which proliferates faster. A total of three biological replicates were performed and displayed as the mean. Error bars represent s.d. Student's *t*-test was performed and significance was found when *P*≤0.05 (marked by *). (F) Expression of neural stem cell markers reveals that the cells have a neural progenitor phenotype. Immunocytochemistry was performed for VIM, NES, SOX2, GFAP and TUBB3. Magnification: 40×; scale bar: 100 µm. (G) Expression of *PAX6* in the RGs revealed increased expression in the hAPPsw^+/+^ lines. Student's *t*-test was performed and significance was found when *P*≤0.05 (marked by *). (H) Expression of *MSI1* in hAPPsw lines did not differ significantly. (I) RGs co-express the radial glial marker BLBP but are negative for the expression of DLX2 (a marker of neuroblasts from the subventricular zone). Magnification: 40×; scale bar: 100 µm. (J) Cell counting of the proportion of BLBP^+^ and GFAP^+^ cells reveal that most cells are double-positive for BLBP/GFAP in the WT and APPsw RGs. A larger proportion of single GFAP^+^ cells are found in the APPsw^+/+^ R5 cell line compared with the other cell lines.

Production of a stable *in vitro* neural cell system in a large mammal model, which carries a genetic background of familial AD, would be of significant interest for researchers studying cellular mechanisms of the disease. First, we produced ESC cultures from the hAPPsw transgenic minipig. This transgenic model was previously produced by stable transfection of a plasmid cassette containing hAPPsw cDNA preceded by beta-globin sequences to induce splicing and a human PDGFβ promoter in donor cells, which were used in somatic cell nuclear transfer to produce live-born offspring (Kragh et al., 2009). We obtained six embryos following a mating of two hemizygous pigs and mechanically isolated the pluripotent ESC population from the retrieved embryos. Following culture of the isolated stem cells, five of the six (83%) specimens formed outgrowth colonies on mitotically inactivated fibroblasts. Following transfer of small clumps of the outgrowth colonies onto MS5 murine stromal cells, we observed formation of neuronal rosettes from as early as 10 days following co-culture, which were mechanically isolated from day 10 to day 15 of co-culture. A total of six neuronal rosettes were isolated, and four stable cell lines were established in neural medium from four of the cultured neuronal rosettes. See method outlined in Fig. 1A. Of the four generated lines, two were derived from individual neuronal rosettes picked from one embryo background, and the other two lines were also derived from individual neuronal rosettes from another embryo background. The resultant cultured cells were compared in all experiments with a wild-type (WT) cell line that was produced using the same methodology but derived from a healthy age-matched embryo (Rasmussen et al., 2011).

Genotyping by qPCR and evaluation of copy numbers revealed that the two lines derived from the same embryo carried a single copy of the *hAPPsw* mutation and the other two lines from the other embryo carried two copies of the *hAPPsw* mutation (Fig. 1B), therefore confirming that we had produced two hemizygous and two homozygous cell lines. The hemizygous lines were named R1 and R2 and the homozygous lines R4 and R5 after the rosette number they were derived from. To evaluate this finding further, we performed expression analysis of *hAPPsw* in the established R1 and R4 cell lines and compared the expression levels with skin fibroblasts obtained from hemi- and homozygous pigs (Fig. 1C). There was an approximately fourfold difference in the expression of *hAPPsw* between both the R4 homozygous and R1 hemizygous fibroblasts, and an approximately fivefold difference in the RGs. Endogenous *APP* and *Neomycin* expression was also performed in the rosette-derived cells compared with WT and also in fibroblasts derived from a single hemizygous pig (Fig. 1B). Endogenous *APP* was consistent in expression across all cells analyzed and *Neomycin* was predictably expressed only in the transgenic cells. Expression of *hAPPsw* is driven by a human *PDGFβ* promoter; it was therefore important to determine whether these cells expressed *PDGFβ.* Analysis of the rosette-derived cells and their GFAP^+^ differentiated progeny showed that they expressed *PDGFβ* (Fig. 1D). We differentiated WT rosette-cells spontaneously for 21 days and analyzed endogenous expression of *PDGFβ.* We found that expression could be determined in the progenitors as well as in spontaneously differentiated cells. Expression was significantly higher in the undifferentiated and differentiated progenitors than in control porcine fibroblasts. Together, the positive expression of *hAPPsw*, *neomycin* and *PDGFβ* could verify the expression of the construct in our cell lines.

In order to assess the identity of the established cell lines, a cell proliferation assay, immunocytochemistry and comparative qPCR were performed. The cell proliferation assay revealed that the cells grew at a rate of 2.8-13.57 population doublings within six days (Fig. 1E). The WT rosette-derived cell line had an average of six doubling rates after six days. The R2 hemizygous line had a comparable doubling rate, whereas the hemizygous R1 and homozygous R4 lines had slightly lower population doubling times. The R5 homozygous line had a higher population doubling time than the WT cell line. These cells were able to grow as a monolayer on Matrigel-coated dishes and could be grown for multiple passages in the presence of bFGF and EGF. Immunocytochemistry for VIM, NES, SOX2, GFAP and TUBB3 was performed in addition to comparative qPCR for *MSI1* and *PAX6*, on all the cell lines and the WT cell lines (Fig. 1F-H). These cell lines expressed VIM, NES, SOX2 and GFAP, and some lines had a cell population which expressed TUBB3 (Fig. 1F). Expression of *PAX6* was found to be significantly higher in both the homozygous hAPPsw^+/+^ lines compared with the hemizygous lines (Fig. 1G). A low expression level of *MSI1* was also observed across all cell lines (Ct range: 34.3-38.8), with little differences in expression between the cell lines (Fig. 1H). Together, these results indicated that we produced neural progenitor cells similar to those we created in a previous study using the same protocol (Rasmussen et al., 2011).

To define the identity of the cells further, we tested whether these progenitors were potentially stem cells representative of either the subventricular zone (SVZ) or the subgranular zone (SGZ). RGs, also known as Type 1 cells from the SGZ, express Nestin, Gfap and Blbp, whereas slightly more differentiated type 2/2a cells lose Gfap and Blbp expression (Steiner et al., 2006; Morrens et al., 2012). Furthermore, B cells from the SVZ express both Nestin and Gfap but A cells (proliferating neuroblasts) express more mature markers, such as Tubb3 and Dlx2 (Ghashghaei et al., 2006; Morrens et al., 2012). We therefore performed co-labeling expression analysis on the WT and hAPPsw lines for GFAP/BLBP, which identify Type 1 cells, and also for TUBB3/DLX2 to identify Type B or Type A cells. Expression of DLX2 only was found to be weak in all the lines, even in the TUBB3^+^ cells; we therefore assumed that these cells were dissimilar to Type A proliferating neuroblasts from the SVZ (Fig. 1I). Expression of BLBP, however, was detected across all cell lines (Fig. 1I). In the WT cells, the majority co-expressed GFAP and BLBP, with only a small proportion exclusively expressing BLBP (Fig. 1I,J). The hemi- and homozygous cells expressed a larger percentage of BLBP^+^ cells but also a sub-population of only GFAP^+^ cells (Fig. 1J). This suggests that these cells have an expression profile similar to Type 1 cells from the SGZ. We therefore considered these cells to be similar to radial glia, and termed them radial glial-like cells (RGs). Chromosomal analysis was also performed on all lines by counting of chromosome spreads. We observed that the R2 cell line had an abnormal karyotype of >38 chromosomes (data not shown). This line was therefore omitted from all subsequent experiments.

### The hAPPsw RGs form astrocytes upon directed differentiation

**Fig. 2.**
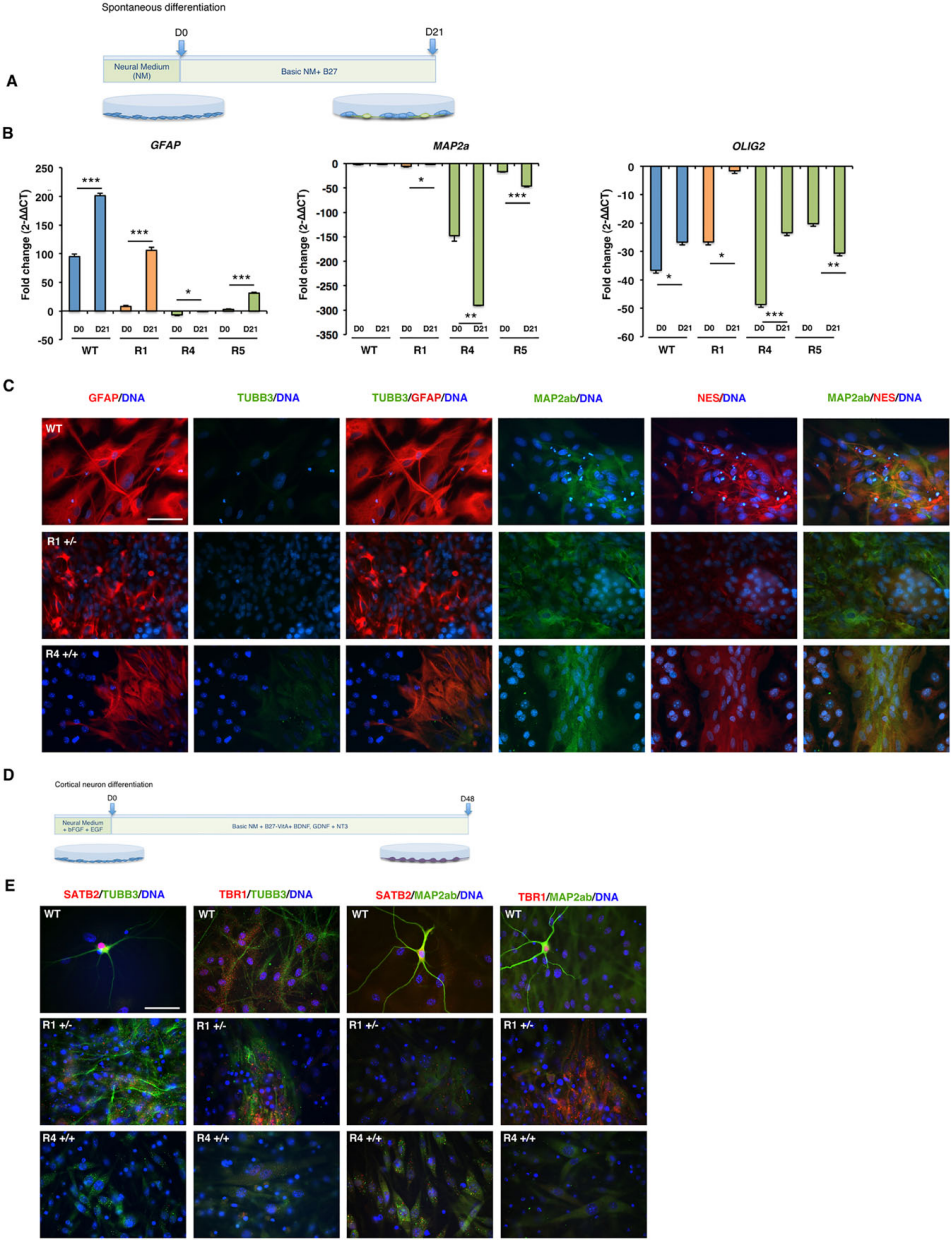
***In vitro* differentiation of RGs results in astrogenesis but not neurogenesis.** (A) Overview of the protocol used to differentiate spontaneously the RGs into neural cells. Neural medium (NM). (B) Expression of *GFAP*, *MAP2a* and *OLIG2* in spontaneously differentiated RGs reveal increased *GFAP* expression following differentiation in both WT and APPsw lines. Error bars represent s.d. Student's *t*-test was performed and significance was found when *P*≤0.05 (marked by *), *P*≤0.01 (marked by **), *P*≤0.001 (marked by ***). (C) Wild-type RGs form astrocytes and neurons, whereas hAPPsw form only astrocytes. Immunocytochemical analysis of GFAP, TUBB3, MAP2ab and NES revealed the WT cells expressed high levels of the astrocyte marker GFAP, but little to no TUBB3, and a minor population of cells were MAP2ab^+^. The APPsw RGs only upregulated GFAP. Magnification: 40×; scale bar: 100 µm. (D) Overview of the protocol used to differentiate the RGs into cortical neurons. (E) Expression of immature and mature cortical neurons was found in the differentiated WT RGs but not in the hAPPsw RGs. Co-localization of either SATB2 with TUBB3 and MAP2ab or TBR1 with TUBB3 and MAP2ab was performed. Magnification: 40×; scale bar: 100 µm.

Following the analysis of the neural markers, we analyzed their ability to form more specific neural cell types by *in vitro* differentiation by performing several different differentiation protocols, as we have been able to previously generate neurons, oligodendrocytes and astrocytes from the WT cell line (Rasmussen et al., 2011). First, we performed a spontaneous differentiation for 21 days in which bFGF and EGF were removed from the culture (Fig. 2A). We analyzed the differentiated cells for expression of the residual progenitor markers *SOX2*, *VIM* and *MSI1*, of the neuron marker *MAP2α*, the oligodendrocyte markers *OLIG2* and *MBP*, and the glial marker *GFAP*. The homozygous line R5 showed downregulation of the analyzed progenitor markers, but only *MSI1* was downregulated in the homozygous line R1, and no significant downregulation of the progenitor markers was evident in the WT line (data not shown), which indicated that neural progenitor markers were expressed at a steady level despite attempts of differentiation in some lines. By contrast, analysis of *GFAP* expression revealed that all lines had increased expression after differentiation (Fig. 2B). Upon analysis of GFAP by immunocytochemistry, a large proportion of the cells expressed this marker and displayed abundant cytoplasmic processes (Fig. 2C), indicative that astrocytes could form in culture. Investigation of neuron markers, including *MAP2a* failed to show that the cell lines could form mature neurons under spontaneous differentiation (Fig. 2B). Expression of MAP2ab revealed some positive signal present in varying populations within all lines, although the morphology of the cells failed to confirm a mature neuron phenotype (Fig. 2C). Investigation of the immature neuron marker TUBB3 did reveal that the WT cell lines contained some positive cells; however, those that were positive in the hemizygous and homozygous lines looked preneural in morphology (Fig. 2C). To investigate the differentiation ability of the cell lines further, we analyzed the expression of oligodendrocyte precursor marker *OLIG2* and the more mature marker *MBP.* In both the WT, R1 hAPPsw^+/−^ and R4 hAPPsw^+/+^ line, an increase of *OLIG2* was observed (Fig. 2B). However, this was not the case for the R5 hAPPsw^+/+^ line. In the case of *MBP*, an increase in expression was only observed in the APPsw^+/+^ lines but this remained more than 200-fold lower than expression in the fetal brain (normalized control) (data not shown). Thus, spontaneous differentiation could result in the generation of the increase in the expression of oligodendrocyte progenitors in some lines and markers of more mature oligodendrocytes in others; however, expression levels remained lower than in the fetal brain controls. Spontaneous differentiation, however, did not lead to significant neurogenesis in any of the lines; therefore, we attempted to induce specific cell types, such as cholinergic neurons, motor neurons or oligodendrocytes, using more targeted differentiation protocols.

First, we performed directed differentiation of the RG lines over 21 days using a combination of sonic hedgehog (SHH), Vitronectin and ciliary neurotrophic factor (CNTF), with the hope of inducing a cholinergic neuron-like phenotype. Comparative qPCR and immunocytochemical analysis was performed, and analysis of the same markers by qPCR revealed that an increase in expression of *GFAP*/GFAP occurred. However, we were unable to detect either an upregulation in the neuronal genes *MAP2a*, *NF*, *CHAT* or of *OLIG2* by qPCR (data not shown). Analysis of the cholinergic marker CHAT revealed a small population of positive CHAT neurons in the differentiated WT RGs (data not shown). We therefore performed two additional differentiation protocols in an attempt to induce both motor neurons and oligodendrocytes based on our previous publication (Rasmussen et al., 2011). Both protocols were unable to yield either neurons or oligodendrocytes (data not shown). We considered that the timing of differentiation could be a crucial factor. Therefore, we employed a longer differentiation protocol based on the recent article reporting high efficient production of cortical neurons from human induced pluripotent stem cells (Kondo et al., 2013). Here, we employed a 48-day differentiation protocol using a combination of different factors, specifically, BDNF, GDNF and NT3 (Fig. 2D), to differentiate WT, R1 hAPPsw^+/−^ and R4 hAPPsw^+/+^ RGs. We analyzed the cells at the end of the protocol for cortical neuron markers both in immature and mature neurons by co-localization with either TUBB3 or MAP2ab, and found that the WT RGs formed both immature neurons that were positive for SATB2 and mature neurons that were positive for both TBR1 and SATB2 (Fig. 2E). However, we were only able to detect weak expression of TUBB3 and MAP2ab in the R1 hAPPsw^+/−^ and R4 hAPPsw^+/+^ RGs, and no labeling of TBR1 or SATB2 could be detected. Together, these differentiation studies suggested that we could produce both astrocytes and neurons from the WT RGs; however, all hAPPsw RG lines were more difficult to differentiate *in vitro* into neurons and only formed astrocytes in culture. Reactive astrocytes play a main role in the inflammatory response in AD (Kitazawa et al., 2004) and have recently been shown to play a key role in early events in the formation of amyloid plaques (Reyes et al., 2008), as well as to have impaired APP processing and secrete Aβ (Zhao et al., 2011). We therefore thought it might be of interest to study AD pathology in the spontaneously differentiated APPsw RGs.

### Impaired APP-related secretase expression is observed in hAPPsw RG-derived astrocytes

**Fig. 3.**
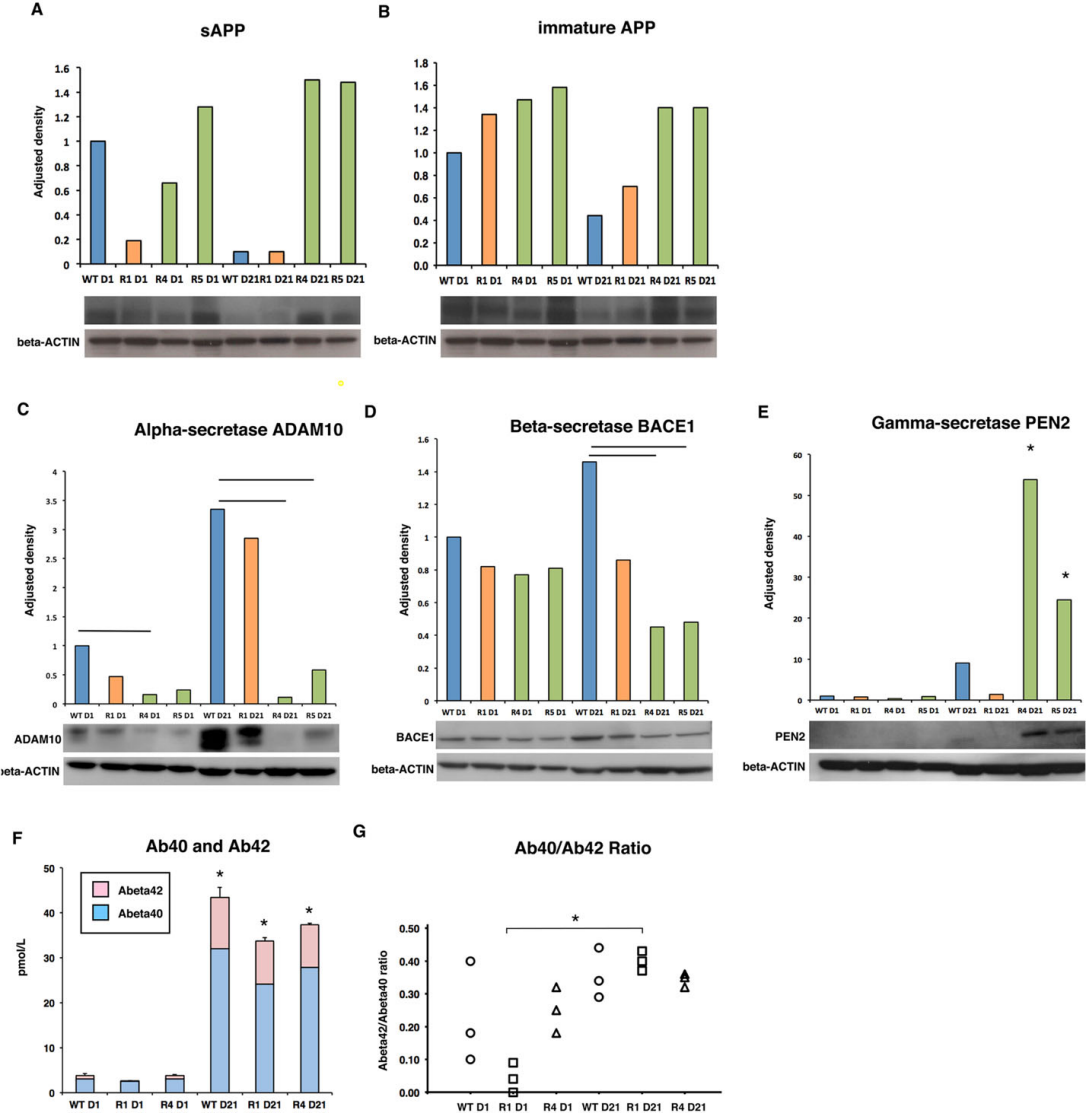
**Altered APP-related secretase expression occurs in hAPPsw^+/+^ RG-differentiated astrocytes but does not lead to an increase in Aβ production.** (A) Western blot using the anti-APP(A4) revealed a significant decrease in sAPP following differentiation of WT RGs but no change in sAPP in the hAPPsw RGs. Expression differences are shown as adjusted density following normalization to β-Actin. A typical western blot is shown. Error bars denote s.d. (B) Western blot using the anti-APP(A4) revealed the presence of immature APP in both WT and hAPPsw RGs. A typical western blot is shown. Error bars denote s.d. (C) ADAM10 α-secretase expression is decreased in both undifferentiated hAPPsw^+/+^ RGs and in astrocytes. A typical western blot is shown. Student's *t*-test was performed on biological replicates and significance was found when *P*≤0.05. Error bars denote s.d. (D) BACE1 β-secretase expression is decreased in hAPPsw^+/+^ astrocytes compared with WT astrocytes. A typical western blot is shown. Student's *t*-test was performed on biological replicates and significance was found when *P*≤0.05. Error bars denote s.d. (E) PEN2, γ-secretase expression is increased in hAPPsw^+/+^ differentiated RGs compared with WT RGs. A typical western blot is shown. Student's *t*-test was performed on biological replicates and significance was found when *P*≤0.05 (marked by *). Error bars denote s.d. (F) Levels of secreted Aβ40 and Aβ42 peptides increase during differentiation into astrocytes but are not significantly different between hAPPsw and WT cells. Student's *t*-test was performed on biological replicates and significance was found when *P*≤0.05 (marked by *). Error bars denote s.d. (G) The Aβ40:Abβ2 of secreted peptides increased in hAPPsw^+/−^ astrocytes compared with undifferentiated hAPPsw^+/−^ astrocytes but this was not significantly higher than WT astrocytes. Student's *t*-test was performed on biological replicates and significance was found when *P*≤0.05 (marked by *).

Considering that previous research has shown that fibroblasts from patients carrying the APPsw mutation and human neuroblastoma cells overexpressing the mutation secrete higher levels of Aβ (Cai et al., 1993; Citron et al., 1994), we investigated APP cleavage in the astrocyte populations. We first tested which forms of APP could be detected in the hAPPsw RGs and their differentiated progeny. Western analysis using an antibody raised against the N-terminus of the precursor APP A4 protein revealed that the cells expressed both soluble APP (sAPP) (a band was detected at 120 kDa) and immature APP (band present at 110 kDa), but not mature APP (Fig. 3A,B) (expected band at 130 kDa). Interestingly, both sAPP and immature APP were more highly expressed in the differentiated hAPPsw astrocytes. The lack of expression of mature APP was not surprising, as these RGs failed to produce mature neurons. We then investigated α-, β- and γ-secretase expression by western analyses. In the undifferentiated RGs, α-secretase expression was, surprisingly, significantly decreased in the hAPPsw^+/+^ R4 line when compared with the WT RGs, and a trend in decrease was observed in the R1 hemizygous and R5 homozygous lines (Fig. 3C). Following spontaneous differentiation, α-secretase remained significantly lower in both hAPPSw^+/+^ lines compared with the hAPPsw^+/−^ and WT RGs, and a lower trend was observed in the hAPPsw^+/−^ differentiated RGs (Fig. 3C). We detected no significant differences in the expression of BACE1 between the WT and hAPPsw RGs (Fig. 3D). Following differentiation, BACE1 was significantly decreased in the hAPPsw^+/+^ astrocytes compared with the WT astrocytes (Fig. 3D). Evaluation of γ-secretase expression was performed by western analysis of PEN2. No difference in gamma expression was observed in the undifferentiated RGs; however, a significant increase in PEN2 was observed in the differentiated hAPPsw^+/+^ glia when compared with WT and hAPPsw^+/−^ glia (Fig. 3E). Together, these data show that the hAPPsw^+/+^ RGs had significantly reduced α- and β-secretase expression, yet increased γ-secretase expression.

We then analyzed the extracellular secretion of small Aβ peptides by performing ELISA to assess the level of secreted peptides Abeta40 (Aβ40) and Abeta42 (Aβ42) in the undifferentiated versus spontaneously differentiated hAPPsw RGs. We observed that a significant increase in both Aβ40 and Aβ42 could be determined following differentiation of the WT and RG-differentiated astrocytes, although levels were comparable in the differentiated cells between both the WT and APPsw cells (Fig. 3F). Although a trend was noted for lower levels of total secreted Aβ40 for both the hAPPsw^+/−^ and hAPPsw^+/+^, this was not found to be significantly different from the WT cells (Fig. 3F). No difference was observed in the level of Aβ42 between the cells (Fig. 3F). Analysis of the Aβ42/Aβ40 ratio showed no difference between the hAPPsw RGs and WT RGs, either when undifferentiated or spontaneously differentiated into astrocytes (Fig. 3G). Although a significant increase in the Aβ42/Aβ40 ratio was observed following differentiation of the hAPPsw^+/−^ RGs, this ratio was not significantly different when directly compared with either hAPPsw^+/+^ differentiated hAPPSw or WT differentiated RGs (Fig. 3G). Therefore, to summarize, the APPsw astrocytes do not contribute to increased levels of either Aβ42 or Aβ40 peptides.

### Phosphorylated tau is increased and alternately spliced in astrocytes derived from hAPPsw^+/+^ RG

**Fig. 4.**
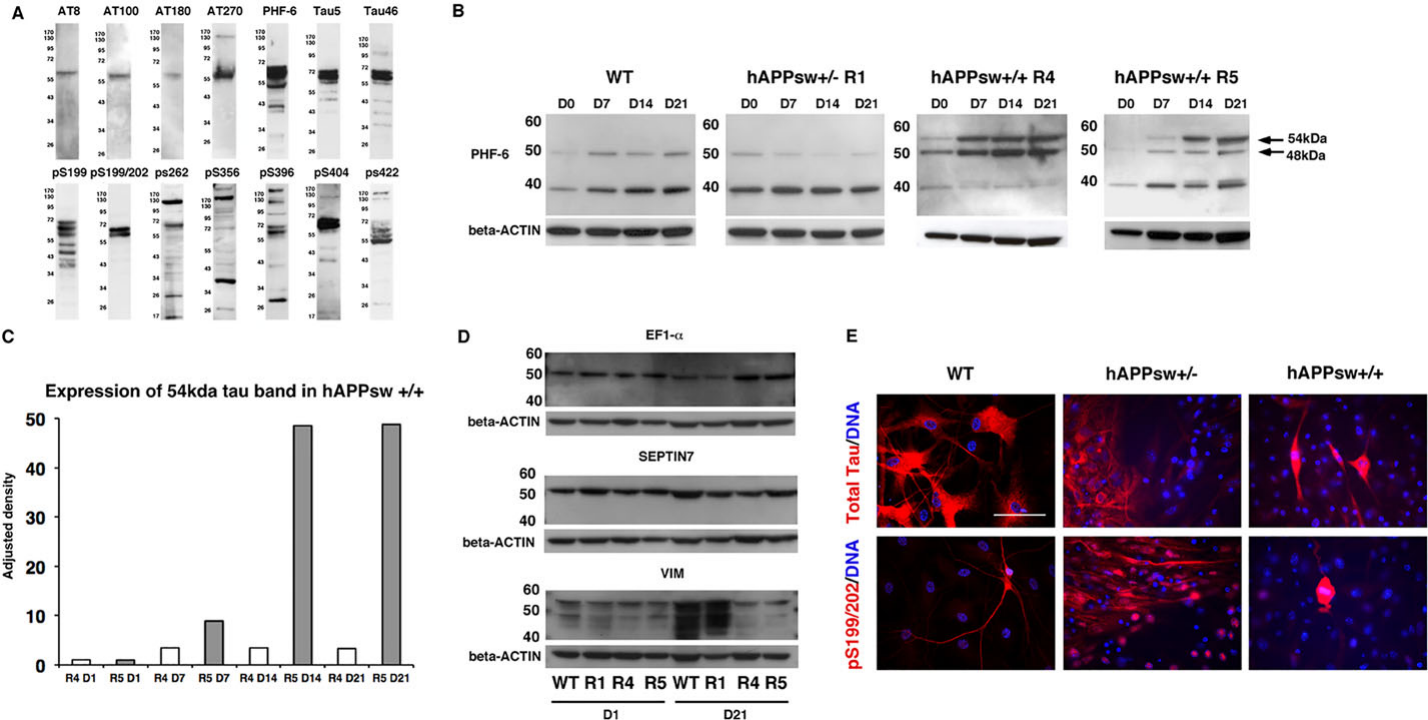
**Altered splicing of tau occurs during differentiation of hAPPsw^+/+^ RGs into astrocytes.** (A) Expression of tau antibodies in porcine adult brain reveals the presence of several isoforms of total and phosphorylated tau. (B) Altered splicing of tau is detected using anti-PHF-6 recognizing phosphorylated tau at Thr^231^ in both hAPPsw^+/+^ cell lines both prior to and during differentiation. An additional band of 54 kDa is observed and increased expression of 48 kDa is observed in the R4 hAPPsw^+/+^ line. (C) Levels of expression of the 54 kDa band reveal a significant increase in the R5 hAPPsw^+/+^ cell line. Adjusted density is calculated by normalization to β-Actin and then adjusted by normalization to R4 hAPPsw^+/+^ undifferentiated RGs. (D) Western blot confirmed that the 54 kDa band of interest is not EF1-α, SEPTIN7 or VIM, which were predicted proteins following mass spectrometry. This was evident as expression of these proteins were detected in the WT and R1 hAPP^+/−^ RGs. (E) Expression of total tau and pSer^199/202^ tau varied between the cell lines and revealed that hyperphosphorylated tau was present only in mitotically dividing hAPPsw^+/+^ cells following a 48-day differentiation into cortical neurons. Magnification: 40×; scale bar: 100 µm.

Given that APPsw animal models have failed to show an increase in phosphorylated tau (p-tau), we aimed to determine whether the astrocytes produced from hAPPsw RGs would reveal any related pathology. We first tested 14 different antibodies, known to react with either mouse or human tau in porcine adult brain. Total porcine tau was analyzed using antibodies Tau5 and Tau46, and p-tau was analyzed using the antibodies AT8 (Ser202/Thr205), AT100 (Ser212/Thr214), AT180 (Thr231/Ser235), AT270 (Thr181), PHF-6 (Thr231), pS199, pS199/202, pS262, pS356, pS396, pS404 and pS422 (Fig. 4A). A single band of ∼60 kDa was observed in porcine adult brain when using AT8, AT100 and AT180 antibodies (Fig. 4A). An additional band was observed at ∼130 kDa in AT270, pS262, pS396, pS404 and pS422. Multiple bands could also be detected, ranging from ∼17 kDa to more than 170 kDa. The high molecular weight bands are probably unspecific. Five antibodies, AT8, PHF-6, Tau5, pS199/202 and pS404, were selected for further analysis and tested in porcine fetal brain, porcine fetal fibroblasts and in undifferentiated and differentiated RGs. Adult porcine fibroblasts expressed a weak band of tau (∼54 kDa) for three of the antibodies (AT8, Tau5, PHF-6) (data not shown). In the case of fetal brain, two isoforms of tau were detected using PHF-6, which were ∼39 kDa and ∼48 kDa in size, respectively (data not shown). A single band >220 kDa was also detected with the AT8 antibody (data not shown). We then evaluated the hAPPsw RGs and RG-derived astrocytes and found no difference in the expression of AT8, Tau5, pS199/202 and pS404 between the hAPPsw RGs and their differentiated progeny compared with the WT RGs (data not shown). In the case of phosphorylated PHF-6, we detected an additional tau band in the spontaneous differentiated hAPPsw^+/+^ lines, which increased in expression during differentiation (Fig. 4B,C). This band corresponded to ∼54 kDa in size. The 48-kDa band was also stronger in the R4 hAPPsw cell line. To confirm the identity of the protein, the 54-kDa band was isolated following Coomassie staining and analyzed by mass spectrometry to confirm whether it was tau specific. Mass spectrometry, however, identified other candidates, including, EF1-α, VIM or SEPTIN7. Therefore, to test the specificity of the western blotting, westerns blots were performed using antibodies raised against all three proteins to confirm the exact identity of the protein band. However, in all three cases, expression of the proteins were identified in all the samples, including the WT and hAPPsw^+/−^ RGs, as well as the differentiated hAPPsw^+/+^ astrocytes (Fig. 4D). Therefore, we were able to exclude these proteins as the source of the novel band in the hAPPsw^+/+^ lines. We have also experienced difficulty in identifying Tau by mass spectrometry in a previous study (Volke and Hoffmann, 2006). It therefore remains a distinct likelihood that the novel band is a specific tau isoform, which could not be identified in mass spectrometry due to strong signals from overlaying proteins, such as EF1-α, VIM and SEPTIN7. The recombinant human tau isoforms have molecular weights of 48-67 kDa, with 3R,0N migrating at 48 kDa, 4R,0N at 52 kDa, 3R,29N at 54 kDa, 4R,29N at 59 kDa, 3R,58N at 62 kDa and 4R,58N at 67 kDa (Goedert and Jakes, 1990). We observe here a probably altered splicing of the Tau in the porcine hAPP^+/+^ cells, giving rise to a band at the same size as the 3R,29N isoform of p-tau. This APPSw model thus recapitulates a probable perturbed splicing of tau.

Next, we tested whether we could identify the expression of tau in RG-derived astrocytes by immunocytochemistry. We first examined expression of total tau and pS199/202 tau in RGs that were differentiated using the cortical neuron differentiation protocol. Total tau was expressed in a large proportion of differentiated WT RGs (Fig. 4E). A lower number of cells expressed total tau in the hAPPsw differentiated RGs, which is probably due to the impaired neurogenesis (Fig. 4E). We also detected expression of pS199/202 tau in a number of WT differentiated RGs and hAPPsw^+/−^ differentiated RGs; however, we were surprised to find that hyperphosphorylation of pS199/202 Tau was detectable in a small number of cells in the differentiated hAPPsw^+/+^ RGs (Fig. 4E). This hyperphosphorylation state appeared to occur only in cells undergoing mitosis, from evaluation of the M phase of the chromosomes by DNA staining. We were not expecting to see dividing cells after the 48 days differentiation protocol and wondered whether this hyperphosphorylation was an event associated with general division, or whether it was specific to the hAPPsw^+/+^ background. We therefore analyzed undifferentiated RGs from the WT and hAPPsw backgrounds further.

### hAPPsw RGs have impaired cell signaling and ribosomal RNA synthesis

**Fig. 5.**
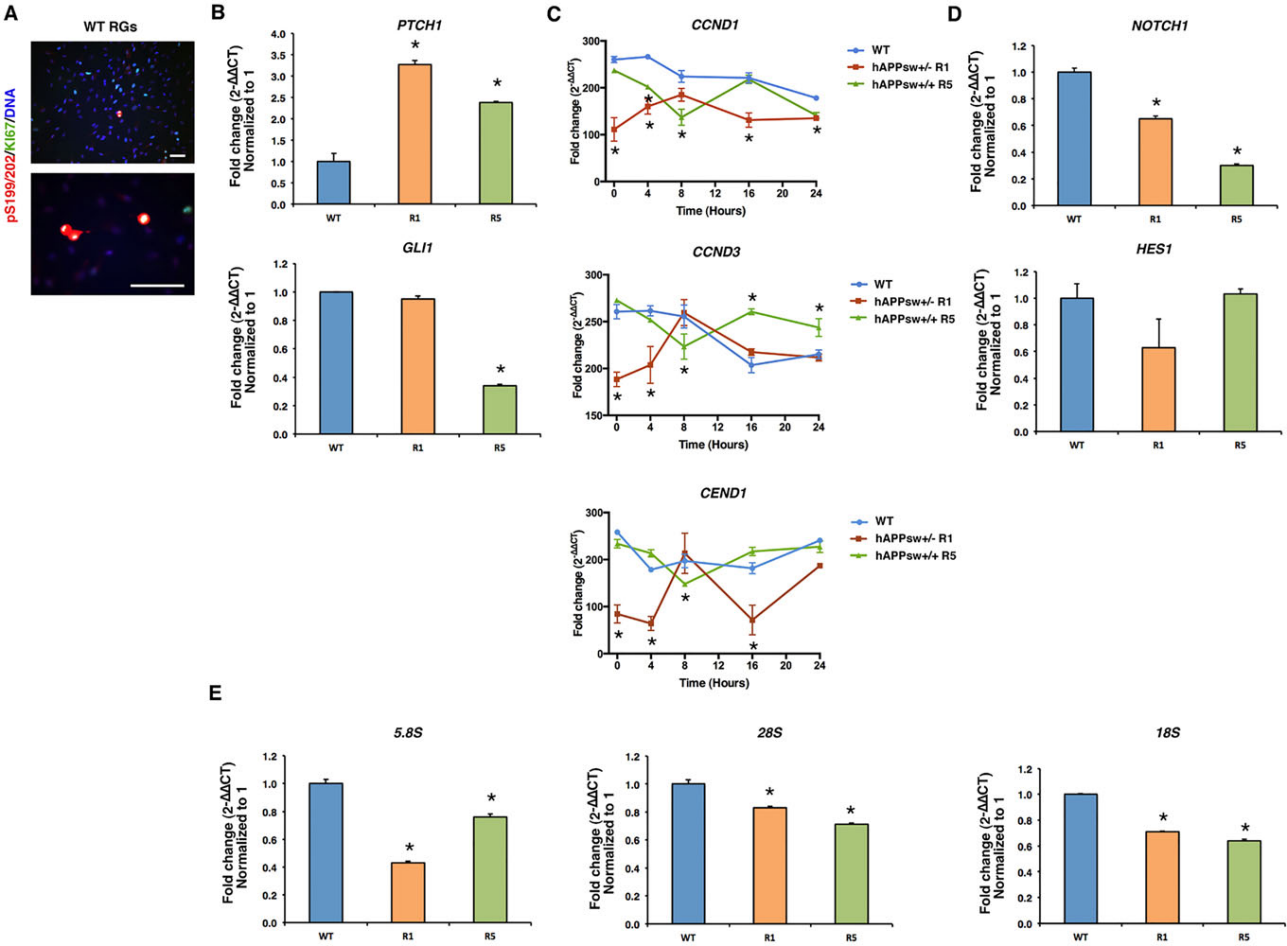
**Genes associated with SHH signaling, the cell-cycle, NOTCH signaling and ribosomal RNA synthesis are dysregulated in hAPPsw RGs.** (A) Expression of hyperphosphorylated pSer^199/202^ tau occurs in conjunction with mitotically dividing WT RGs (KI67^+^) and is therefore a normal process occurring during cell division. Magnification: 16× and 40×; scale bar: 100 µm. (B) Evaluation of the genes involved in SHH signaling show an increased expression of *PTCH1* in hAPPsw RGs; however, the downstream marker *GLI1* is decreased in hAPPsw^+/+^ RGs compared with WT. Student's *t*-test was performed on biological replicates and significance was found when *P*≤0.05 (marked by *). (C) Cell synchronization was performed to assess the expression of Cyclin D genes and CEND1 over 24 h. Cyclin D1 (*CCND1*) was significantly decreased in the hAPPsw RGs and altered expression of Cyclin D3 (*CCND3*) was observed at varying time points for the hAPPsw RGs. *CEND1* was mostly decreased in the hAPPsw^+/−^ RGs compared with WT RGs. Student's *t*-test was performed on biological replicates and significance was found when *P*≤0.05 (marked by *). (D) Expression of NOTCH signaling genes revealed a decrease in *NOTCH1* in the hAPPsw RGs but no change was observed in expression of *HES1*. Student's *t*-test was performed on biological replicates and significance was found when *P*≤0.05 (marked by *). (E) Expression of the ribosomal RNA subunits 5.8S, 18S and 28S was significantly reduced in hAPPsw RGs compared with the WT RGs. For all comparative qPCR, Student's *t*-test was performed on three biological replicates and significance was found when *P*≤0.05 (marked by *).

We first performed ki-67 co-labeling with pS199/202 and detected in the WT undifferentiated RGs that ki-67 staining was localized to the same cells that had hyperphosphorylated pS199/202 tau (Fig. 5A). This demonstrated that hyperphosphorylation of tau was occurring coincidentally with cell division within the progenitor cell pool. It has been previously shown that hyperphosphorylation of tau can occur in both human and murine neuroblastoma cell lines (Preuss and Mandelkow, 1998), as well as in other cell types such as Chinese hamster ovary cells, particularly during metaphase (Illenberger et al., 1998). We were, however, perplexed to see that dividing cells were still present after long differentiation protocols and only in the hAPPsw differentiated RGs. Thus, in order to investigate cell cycle dynamics, we performed qPCR analysis for genes involved in sonic hedgehog (SHH) signaling, namely *PTCH1* and *GLI1*, and genes involved in the cell cycle, *CCND1* and *CCND3*, and in cell cycle exit, *CEND1*. Interestingly, expression of the receptor *PTCH1* was significantly increased in the hAPPsw RGs (Fig. 5B), and *GLI1* was significantly reduced in the hAPPsw^+/+^ RGs, but not in the hAPP^+/−^ RGs compared with the controls (Fig. 5B). GLI1 is transported to the nucleus and activates several genes, including Cyclin D1 (*CCND1*) and Cyclin D3 (*CCND3*). Therefore, we performed a time-course assessment over 24 h for the expression of *CCND1*, *CCND3* and *CEND1*, the important marker of co-ordination of cell cycle exit and differentiation of neural progenitors (Politis et al., 2008), by inducing cell synchronization by growing cells to confluency (cell cycle phase G0/G1). We found that *CCND1* was downregulated at almost all time points during the hAPPsw RG cell cycle compared with WT (Fig. 5C). In addition, dysregulation of CCND3 expression was confirmed for the hAPPsw RGs at several phases of the cell cycle compared with the WT controls. This confirmed that dysregulation of Cyclin D activity might be directly coupled with altered *PTCH1*-*GLI1* signaling in the hAPPsw RGs. Analyses of *CEND1* expression revealed that expression was significantly reduced at almost all time points for the RG hAPPsw^+/−^ cells but not for the hAPPsw^+/+^ RGs (Fig. 5C). This decrease might help to explain partly an impaired neurogenesis in the cells due to a possible lack of exit from the cell cycle.

NOTCH signaling is directly involved in APP-induced glial differentiation. The NOTCH receptors are also directly cleaved by γ-secretase (Niwano and Aizawa, 1991), which results in production of NICD, thus inducing expression of downstream factors such as HES1. Given that we observed an upregulation of the PEN2 γ-secretase in the hAPPsw RGs, we studied this pathway. Based on qPCR results, we found that *NOTCH1* expression was significantly reduced in the hAPPsw RGs (Fig. 5D). However, expression of *HES1* was not altered in the APPsw RGs (Fig. 5D). Whether the downregulation of *NOTCH1* observed in the hAPPsw is of importance for RG function remains to be determined.

Finally, in order to determine whether the APPsw RGs displayed any other early features of the disease, we decided to analyze ribosomal gene expression, as altered ribosomal synthesis and processing have been observed in AD (Ding et al., 2005). We analyzed expression of the ribosomal RNA subunits 5.8S, 18S and 28S in the RGs and found that these were all significantly reduced in the hAPPsw RGs (Fig. 5E), indicating a reduction in basic RNA synthesis in early progenitors. In summary, we found perturbations in both cell signaling and ribosomal synthesis in the hAPPsw RGs, which indicate basic cellular defects to be present at very early stages of neuronal development.

## DISCUSSION

Here, we describe production of porcine cells, which we termed RGs, expressing markers typically observed in Type 1 cells observed in the SGZ, a region in the hippocampus where adult neurogenesis occurs. The high co-expression of BLBP and GFAP in these cells suggests that these were ideal progenitors for analysis into early defects in hippocampal neurogenesis of AD. Given that several new pig models have been produced for studying neurodegeneration showing great promise for this field (Dolezalova et al., 2014), we also considered that production of a pig model carrying a familial AD mutation (APPsw) could help to provide further insight into the disease compared with current similar rodent models. In addition, the pig has highly homologous AD-related genes (PSEN1, PSEN2) compared with the human (Madsen et al., 2007), shows better neuropathology in the brain compared with rodent models when used as a model for the neurodegenerative Huntington's disease (Li and Li, 2012), and its brain has close anatomical and physiological similarities to the human brain (Jelsing et al., 2005). Production of the RGs was based on previously established protocols using MS5 bone marrow stromal cells to induce differentiation of neural rosettes from pluripotent stem cells (Lee et al., 2007, 2009; Dubois-Dauphin et al., 2010; Rasmussen et al., 2011). Taken together, we have produced a unique *in vitro* RG model that gives us further insight into familial AD-related mechanisms years before the disease pathology might emerge in the transgenic animal model itself.

From the performance of several different differentiation protocols, we found that impaired neurogenesis was evident in the hAPPsw RGs. Reduced neurogenesis has been demonstrated in the adult AD brain (Mu and Gage, 2011) and in several mouse models of AD (Rodríguez et al., 2008; Demars et al., 2010; Faure et al., 2011), and is considered to be an early event of the disease. Increased astrogenesis has also been observed in the hippocampus of a mouse PSEN1 AD model and in the neocortex of APP23 mice (Eder-Colli et al., 2009; Bondolfi et al., 2002). This neurogenic impairment was neither related to lower proliferation rates, which have been reported in the APPswe/PS1DeltaE9 mouse (Demars et al., 2010), nor do we believe this to be a result of long-term culture of the cells, as we applied all differentiation protocols and analyses on cells cultured for less than ten passages. Instead, we saw impairments in the expression of varying genes related to the cell cycle. In particular, the increased expression of PTCH1 was interesting, which has also been observed in the hippocampus of 12-month-old transgenic APP23 mice (carrying APP Swedish mutation) (He et al., 2014). Interestingly, this same study reported that deficits in PTCH/GLI signaling led to asymmetric cell division, resulting in production of increased transit-amplifying cells and neuroblasts in the hippocampus of the APP23 mice and in increased astrogenesis. This, together with the perturbations in the Cyclin D genes, might be why we see impaired neurogenesis in the hAPPsw RGs. The increased astrogenesis, however, provides us with a unique model for studying the white matter and its role in familial AD, which is often forgotten in research. However, it is particularly important, as astrocytes outnumber neurons fivefold in the brain and become activated in AD, as well as associate closely with neuritic plaques (Mrak and Griffin, 1996).

Very little is known about the contribution of astrocytes to Aβ production in AD or how Aβ might be generated in these cells (Zhao et al., 2011). We did discover that impaired APP-related secretase expression was evident in the APPsw^+/+^ RGs, including decreased ADAM10 α-secretase expression, increased PEN2 γ-secretase expression and lack of increased BACE1 β-secretase activity. Our data suggest, however, that this altered expression levels did not lead to an increased Aβ production. This is coherent with one report on thorn-shaped astrocytes in AD white matter from the temporal lobe, which did not reveal any abnormal inclusions of Aβ (López-González et al., 2013). In addition, the lack of BACE expression was not unusual, given that BACE activity is also not increased in the white matter in AD brains (Bigl et al., 2000). However, a recent report in BACE null mice suggests that decreased BACE activity can directly induce increased astrogenesis at the expense of neurogenesis (Hu et al., 2013). Levels of Aβ40 and Aβ42 were not increased in the AD astrocytes, but of interest was the general high expression of Aβ42, both in WT and the AD cells. This was an interesting phenotype to uncover in the *in vitro*-produced cells but was also seen in a previous study (Kimura et al., 2006). To conclude, our data reveal altered expression of APP-related secretases and no increase in secreted levels of Aβ40 and Aβ42 in *in vitro*-produced astrocytes, which could be an early feature of familial AD.

Investigation of p-tau at site Thr^231^ in our hAPPsw differentiated astrocytes revealed expression of a probably unique isoform of p-tau compared with the controls. Previous research has shown abnormal p-tau at sites Ser^396^ and Ser^404^ in the brain of a rat model containing APPsw (Folkesson et al., 2007). However, little is known of how tau processing might occur in astrocytes or white matter. Two studies have investigated astrocytes from the AD brain and revealed that astrocytes contain hyperphosphorylated tau and that some include conformation changes of tau (Iwatsubo et al., 1994; López-González et al., 2013). Our research therefore helps to substantiate the notion that increased tau-phosphorylation occurs and can even be observed in an *in vitro* model of AD, which might be useful for further research pertaining to tau processing in this disease.

Our analyses of ribosomal RNA synthesis also highlighted the fact that reduced levels of ribosomal RNA synthesis occurs in the APPsw RGs. RNA oxidation is a known feature of the disease that occurs early on in the disease (Shan et al., 2007; Poulsen et al., 2012). However, previous research has also shown decreased ribosomal RNA synthesis in the AD by decreased sizes of nucleolar organizer regions and decreased expression of the ribosomal RNA 28S subunit in peripheral blood (Payão et al., 1998; da Silva et al., 2000; Donmez-Altuntas et al., 2005), but very little is known pertaining to expression of ribosomal RNA synthesis in brain tissue. The impact of this decreased RNA ribosomal synthesis is unknown. Further analyses are clearly needed to investigate dysregulated ribosomal RNA synthesis in this model and in other neural tissue.

In summary, we have produced a unique *in vitro* RG model carrying either one copy or two copies of hAPPsw from a transgenic pig model that will probably develop the disease in several years. They are a particularly useful cell population for analyzing early features of the disease. Upon induced differentiation, we were able to identify only increased astrogenesis, at the expense of neurogenesis, which is a phenotype that has been reported in some models of AD. The RGs displayed altered expression of cell cycle genes and decreased expression of ribosomal RNA genes. The astrocytes generated from the RGs also revealed novel mechanisms pertaining to APP-related secretase expression, but corroborated lack of BACE1 expression as observed in human AD astrocytes. We also observed increased hyperphosphorylation of tau. Together, this research gives us further insight into early etiology of the disease. It also gives some hope to the use of alternative large mammal models and specialized cell types derived from *in vitro* cultures for recapitulating unique features of familial AD by insertion of only a single mutant gene.

## MATERIALS AND METHODS

### Derivation of RGs from ESCs

RGs were produced by differentiation of *in vitro* cultured pluripotent ESCs obtained from transgenic embryos. Briefly, transgenic Göttingen minipigs carrying a single copy of the hAPPsw were produced by somatic cell nuclear transfer (Kragh et al., 2009). In this transgenic model, hAPPsw is expressed under the control of the human PDGFβ fragment. A single-copy insertion was detected in the intronic sequences of the widely expressed zinc-finger transcription factor *GLIS3*, in the reverse orientation within the genomic DNA of all cloned offspring. High transgene expression has been detected in the cortex, cerebellum, hippocampus, basal ganglia and the brain stem in a hemizygous individual. Mating of a 2.5-year-old F0 hemizygous APPsw female sow and a one-year-old F1 hemizygous APPsw boar was performed over two days. Eight days following initial mating, resultant embryos were flushed from the uteri by surgical flushing. Six embryos were recovered and evaluated morphologically as hatched blastocysts, containing a tight, compact epiblast. Epiblasts were mechanically isolated from the embryos, cultured individually on mitotically inactivated mouse embryonic fibroblasts and allowed to outgrow in KO-DMEM (Invitrogen), containing 10% KSR (Invitrogen), 5% heat-inactivated FBS (Invitrogen), 1× NEAA (Sigma), 1× GlutaMax (Invitrogen), 0.1 mM 2-mercaptoethanol (Invitrogen), 1× penicillin/streptomycin (Sigma), supplemented with human recombinant basic fibroblast growth factor (20 ng/ml) (R&D Systems) and human recombinant Activin A (10 ng/ml) (Prospec). Expanded ESC outgrowths were mechanically cut into small pieces five days following culture and placed as small clumps onto mitotically inactivated (mitomycin C-treated) MS5 murine stromal cells, in DMEM medium containing 15% KSR (Invitrogen), 1× GlutaMax, 1× penicillin/streptomycin (Sigma) to induce neural differentiation. Neural rosette-like structures were identified, disaggregated by gentle pipetting, plated onto Matrigel-coated (BD Biosciences) dishes and cultured in DMEM/F12 (Sigma)+1% B27 supplement (Life Technologies), 1% N2 Supplement (Life Technologies), 1× penicillin/streptomycin, supplemented with human EGF (20 ng/ml) (Prospec) and human bFGF (20 ng/ml) (neural medium) (R&D Systems). Basic neural medium was also used for differentiation, which is as described above minus B27 and the growth factors EGF and bFGF. Cells reminiscent of a neural progenitor cell type were observed to grow as monolayers and were passaged continually upon reaching near confluency.

### *In vitro* differentiation of RGs

Spontaneous differentiation was induced in WT, hemizygous and homozygous hAPPsw porcine progenitors by removal of growth factors bFGF and EGF from the culture. Cells were cultured for 21 days and analyzed for expression by comparative qPCR for neural progenitor markers *SOX2*, *NES*, *VIM* and *MSI1*. Cells were also analyzed for *GFAP* (expressed in both progenitors and astrocytes) and *TUBB3* (expressed in both progenitors and neurons), and for expression of *MAP2a*, *OLIG2* and *MBP*.

Directed differentiation into cholinergic neurons was performed by removal of bFGF and EGF on day one of differentiation, and by replacement of B27 with B27 containing Vitamin A. SHH (500 ng/ml) and Vitronectin (5 µg/ml) were supplemented to the media. Media was replaced every second day thereafter. On day seven, Vitronectin was removed from the media. On day 14, SHH was also removed from the media and CNTF (50 ng/ml) was added. This protocol was selected in an attempt to induce basal cholinergic neurons, based on previous literature specifying growth factors that, among others, specify cholinergic neuron development (Rohrer, 1992; Deguchi et al., 1994; Pons and Marti, 2000).

Directed differentiation into cortical neurons was based on a protocol recently published on differentiation of human induced pluripotent stem cells into cortical neurons (Kondo et al., 2013). Briefly, we replaced the neural medium with neural basal medium (Life Technologies) containing 1% B27 minus vitamin A supplement (Life Technologies)+10 ng/ml human recombinant brain-derived neurotrophic factor (BDNF) (Prospec), 10 ng/ml human recombinant glial cell line-derived neurotrophic factor (GDNF) (Prospec)+human recombinant neurotrophin 3 (NT3) (Prospec), and cultured the cells for 48 days. Medium was changed every second day.

Directed differentiation into motor neurons was performed by adding 1 µM retinoic acid+200 ng/ml SHH+20 ng/ml BDNF+0.2 mM Ascorbic Acid (AA) to basic NM for 14 days, followed by culture in 20 ng/ml BDNF+20 ng/ml GDNF+0.2 mM AA for an additional seven days. Medium was changed every second day. Directed differentiation into oligodendrocytes was performed by adding 20 ng/ml platelet-derived growth factor (PDGF) into basic NM for 21 days. Medium was changed every second day.

### Cell genotyping

The established cell lines were genotyped to determine their genetic background using genomic PCR. Genomic DNA was extracted using the Maxwell 16 LEV Blood DNA Kit (Promega-AS1290). Briefly, 25 ng genomic DNA or 2.5 µl cDNA were used as a template to determine the APP695sw copy number or the relative APP695sw mRNA levels, respectively. Endogenous *APP* and *NEO* expression was also performed using primers directed towards the hAPPsw (see supplementary material Table S1). The templates were mixed with 7.5 µl master mix (containing 2.0 pmol of each primer set and 5.0 µl SYBR GREEN (Roche-04887352001), giving a total volume of 10 µl. The mixture was pipetted in a 96-well plate. Each genomic qPCR and reaction was performed in two wells to obtain a technical duplicate. Each RT-qPCR was performed in three wells to obtain a technical triplicate. The qPCR plate was run on the iCycler Thermal Cycler (Bio-Rad). Cycle conditions were: 95°C, 10 s; 60°C, 20 s; 72°C, 30 s; 40 repeats. The APPsw695 cycle number was normalized to *GLIS* 3 representing two copies. The levels of mRNA were normalized to the geometric mean of *TBP* and quantified using the x_0_ method (Thomsen et al., 2010). The *NEO* and APP695sw primers have been previously described (Jakobsen et al., 2012). The TBP reference primers have also been previously validated (Nygard et al., 2007)

### Cell proliferation assay

Cells were characterized by performing a cell proliferation assay, immunocytochemistry (Rasmussen et al., 2011) and comparative qPCR. A previously established porcine epiblast-derived neural progenitor cell line (Rasmussen et al., 2011) was used as a comparative control, which was established using the same method. For the cell proliferation assay, 50,000 cells were plated for each cell line in triplicate for each time point measurement (passage 8-14) and cultured for up to five days. Population doublings were scored daily, for each cell line.

### Cell synchronization assay

The WT and APPsw (R1 and R5) RGs were synchronized to G0/G1 by growing to complete confluency. Cells were then dissociated in accutase and plated at 5000 cells/cm^2^. Cells were harvested at 0 h, 4 h, 8 h, 16 h and 24 h for RNA extraction and comparative qPCR analysis.

### Chromosomal analysis

Metaphase spreads were prepared by culturing RGs in 50 µg/ml colchicine for 1 h. The cells were removed by scraping them from the well and incubating them in hypotonic solution (0.56% KCl) for 20 min at 37°C. The supernatant was removed and cells were fixed in 3:1 of methanol (MeOH)/glacial acetic acid for 30 min. The cells were centrifuged and re-fixed in 3:1 MeOH/glacial acetic acid twice more. Fixed cells were dropped and allowed to spread onto clean glass slides at 60°C. Fresh MeOH/glacial acetic acid was dropped onto the slides and slides were dried at room temperature. The slides containing metaphase spreads were stained for G and C bands by incubation in 2× SSC at 60°C for 1.5 h, then rinsed briefly twice in 0.9% NaCl at room temperature. Slides were then stained for 4-6 min in Trypsin-Giemsa solution (2.2% Giemsa plus 0.0125% trypsin), rinsed in 1:1 PBS and distilled water, and blow-dried with an air jet. Slides were sealed with a coverslip in mounting medium and analyzed at 100× magnification using bright-field microscopy.

### Immunocytochemistry

Immunocytochemistry was performed to target several different antibodies (see supplementary material Table S2). For immunostaining, cells were grown *in vitro* on glass coverslips pre-treated with poly-L-Lysine (Sigma), Laminin (Sigma) and Matrigel (BD Biosciences). Cells were fixed with 4% paraformaldehyde for 15 min at room temperature and stored in PBS at 4°C short term. Cells were permeabilized using 0.1% Triton X-100 in PBS for 1 h at room temperature. Blocking was performed by adding 5% Normal Donkey Serum (Sigma) in PBS (blocking buffer) to the cells and cultured for 1 h at room temperature. Immunolabeling with the primary antibody was performed overnight. Antibodies used and their dilutions are listed in supplementary material Table S2. Cells were rinsed three times using PBS and cultured in secondary fluorescent-conjugated antibodies (Jackson ImmunoResearch) at 1:200 dilution in blocking buffer. Cells were labeled with Hoechst (5 µg/ml). As a negative control, cells were cultured in the absence of the primary antibody and also with a matching isotype instead of the primary antibody. Cells were visualized using a DMRB fluorescent microscope (Leica Microsystems) and a TCS SPE confocal microscope (Leica Microsystems). Images were captured using Leica Application suite (2.8.1) capture and MM-AF software (Leica Microsystems).

### Comparative qPCR

RGs were analyzed for the expression of a multitude of neural and progenitor markers by comparative qPCR. All primers used are listed in supplementary material Table S1. Cells were harvested and lysed in RLT lysis buffer (Qiagen), snap-frozen in liquid nitrogen and stored at −80°C until use. Total RNA was isolated using the RNeasy mini kit (Qiagen) according to the manufacturer's instructions. Fifty nanogram total RNA was amplified using QuantitTect whole transcriptome kit (Qiagen) using the longer amplification cycle (8 h) for spontaneous differentiated cells, whereas fibroblast and RG RNA was converted into cDNA using the RevertAid first strand cDNA synthesis kit (Thermo Scientific). Ten-microliter qPCR reactions were set up and run according to the LightCycler 480 SYBR Green 1 Master manual and were performed in three biological replicates. Standard curves were performed for all primers prior to analysis and melting curves were analyzed for clean single peaks.

### Western blot

Western blot was performed using a mini-gel apparatus system (Biorad) and using reagents from the rabbit and mouse WesternBreeze chemiluminescent kits (Invitrogen), according to manufacturer's instructions. Total protein, including phosphorylated protein, was prepared by homogenization in Radio Immuno Precipitation Buffer (RIPA) lysis buffer containing complete protease inhibitor cocktail (Roche Diagnostics, Basel, Switzerland), followed by incubation for 30 min with constant agitation at 4°C. The lysate was centrifuged at 4°C at 1200 rpm for 20 min and supernatants were transferred and stored at −80°C. For each sample/loading replicate, a total of 10-20 µg of protein was added to NuPAGE LDS sample buffer (Invitrogen), containing NuPAGE Sample Reducing Agent (Invitrogen), and denatured at 70°C for 10 min. Samples were loaded into ready-made 10% Tris-HCl gels (Bio-Rad). Loading markers used were either the MagicMark XP western protein standard (Invitrogen) or the PageRuler prestained protein ruler (Fermentas), and run at 200 V constant for 50 min in NuPAGE MOPS SDS Running Buffer (Invitrogen), containing NuPAGE Antioxidant (Invitrogen). Proteins were blotted on PVDF membranes (GE Healthcare) for 30 V constant for 1 h in NuPAGE Transfer buffer (Invitrogen) containing methanol (Sigma-Aldrich) and NuPAGE antioxidant (Invitrogen). Chemiluminescence immunodetection was performed according to instructions from the WesternBreeze kit (Invitrogen). Antibodies were diluted according to supplementary material Table S2 and incubated overnight on a rotator at 4°C. Membranes were developed using Amersham Hyperfilm ECL (GE Healthcare) within a Hypercassette autoradiography cassette (GE Healthcare) and developed using RG X-Ray developer (Champion Photochemistry, Spain). Gels were stripped by incubation in 0.5 M NaOH for 10 min at room temperature with agitation and re-probed using anti-betaACTIN (1:30,000) (A5441; Sigma-Aldrich), which recognized a single band of 42 kDa.

### ELISA

ELISA was performed using the Aβ (1-40) Elisa kit II (Wako) and the Aβ (1-42) Elisa kit high-sensitive (Wako) according to manufacturer's instructions. The standard curve and biological replicates were performed in triplicate. Supernatant was collected in biological triplicates from undifferentiated and spontaneously differentiated RGs, snap-frozen in liquid nitrogen and stored at −80°C. Upon thawing, samples were centrifuged at 13,000 ***g*** for 10 min at 4°C. ELISA plates were read using a plate reader at 450 nm. A Bradford assay was performed on all protein samples in triplicate and measurements were recorded on the plate reader at 570 nm. Readings from the Bradford protein assay were used to normalize the Aβ values.

### Mass spectrometry

To perform mass spectrometry, a 10% Tris-HCl gel was loaded with 70 µg of protein from differentiated homozygous hAPPsw RGs and run at 200 V constant for 2 h. The gel was stained with Coomassie dye and de-stained in 30% MeOH with a final rinse in 0.5% glacial acetic acid. Bands were cut with an EXQuest Spot Cutter (Bio-Rad Laboratories) and digested as previously described (Volke and Hoffmann, 2006). Tryptic peptides were dissolved in 10 µl 3% aqueous acetonitrile containing 0.1% formic acid and used for a nanoUPLC-Orbitrap-MS/MS. CID spectra were acquired from the three most abundant ions in each MS scan (isolation width 2, activation Q 0.25, normalized collision energy 35%, activation time 30 ms). Conditions and instrument parameters are described elsewhere (Bollineni et al., 2014). The acquired data were analyzed automatically by Sequest search engine (Proteome Discover 1.0, Thermo Fisher), allowing up to two missed cleavage sites and a mass tolerance of 10 ppm for precursor ion scans and 0.8 μ for product ion scans.

### Statistical analysis

In the case of comparative qPCR, statistical analyses were performed on the mean 2^−ΔΔCT^ values, using an independent, unpaired, two-tailed Student’s *t*-test, and significance was determined when *P*≤0.05. Error bars are denoted as s.d. In the case of western blotting of sAPP, immature APP, phosphor APP(pTyr^757^), PHF-6, ADAM10, BACE1 and PEN2, western blot gels were quantitatively assessed using ImageJ gel analysis to produce profile plots. The relative density of the peaks were measured in three separate blots and normalized to relative density of corresponding beta-ACTIN peaks to produce an adjusted density. Differences between treatment groups were assessed using Student's *t*-test as described above. In the case of the ELISAs, the ratio of normalized Aβ42 and Aβ40 values were calculated using Xcell software. Differences between treatment groups were again measured using Student's *t*-test. In the case of the proliferation assay, triplicates were performed and an independent, unpaired, two-tailed Student's *t*-test was used to compare differences between the lines.
